# High HBV Load Weakens Predictive Effect of Serum miR-122 on Response to Sorafenib in Hepatocellular Carcinoma Patients

**DOI:** 10.1155/2021/9938207

**Published:** 2021-06-11

**Authors:** Xiaomin Zhang, Fu'an Wang, Guangfeng Gu, Qingpo Wu

**Affiliations:** ^1^Department of Clinical Medicine, Pingdingshan University, Weilai Road, Xincheng District, Pingdingshan, Henan 467000, China; ^2^Department of Cardiology, The First People's Hospital of Pingdingshan, Youyu Road, Pingdingshan, Henan 467000, China

## Abstract

**Background:**

MiR-122 is a liver-specific microRNA. The aim of the study was to explore the association of serum miR-122 with response to sorafenib in hepatitis B virus- (HBV-) related hepatocellular carcinoma (HCC) patients and to further reveal the effect of the virus load on such potential relationship.

**Methods:**

A total of 588 patients with HCC were retrospectively included. All of them were diagnosed with HBV-related locally advanced HCC and were treated with sorafenib. Therapeutic and prognostic information and other information were collected from medical records. Stored blood specimens that were obtained before sorafenib treatment were adopted to detect miR-122.

**Results:**

The patients were divided into high-level group and low-level group according to the median of serum miR-122 level, and each group contained 294 patients. During the first 24 weeks after sorafenib treatment, the patients in the high-level group had more opportunities to experience progression-free survival (PFS) and overall survival (OS) than those in the low-level group (HR: 2.47, 95%CI: 1.24∼4.88; HR: 1.20, 95%CI: 1.09∼1.32). In the subgroup analysis, the relationship between serum miR-122 level and overall survival still existed in the patients with relatively lower HBV load (HR: 1.22, 95%CI: 1.09∼1.36), but not in the patients with higher HBV load (HR: 1.12, 95%CI: 0.93∼1.35).

**Conclusion:**

Higher serum level of miR-122 at baseline was associated with a better response to sorafenib in HBV-related locally advanced HCC patients, and relatively high HBV load weakened such predictive effect mentioned above.

## 1. Introduction

Hepatocellular carcinoma (HCC) is the dominant type of primary liver cancer in human beings, accounting for about 90% of total cases all around the world [1]. In China, HCC is a very common cancer with an estimated incidence rate of 0.40‰ in males and 0.15‰ in females [2]. Also in this country, the major cause of cancer is hepatitis B virus (HBV) infection, not long-term drinking [2].

HCC is a malignant disease with poor prognosis. Previous studies reported that the expected median survival period of the patients does not exceed five months without treatment [3, 4]. In the last decade or two, diagnostic technology is developing rapidly and allows more than 50% of the patients to be confirmed at a locally advanced stage. This provides a valuable opportunity for early intervention against HCC.

Sorafenib (SOR) is an oral molecule-targeted drug against malignancies which is introduced in 2007. Clinical trials report that SOR significantly increases the median overall survival (OS) of HCC patients [5, 6]. However, not all patients are sensitive to the drug, and the response to SOR has become one of the most important factors affecting the efficacy of SOR and the prognosis of the patients. To date, studies have reported many potential predictors of SOR response, such as proteins, cell-free DNA, microRNA, and inflammatory markers, but their predictive power is unsatisfactory or not fully confirmed [7–9]. Therefore, unlike other targeted therapies, available predictors in HCC patients treated with SOR are lacking, and related research is still ongoing.

MicroRNA-122 (miR-122) is a liver-specific microRNA. Tissue expression of it can be changed in many liver diseases [10]. MiR-122 has also been discovered in peripheral circulation, and an increased level of miR-122 in serum is associated with the progression of viral hepatitis, nonalcoholic fatty liver disease, and other liver injuries [11–13]. During in vitro experiments, downregulation of miR-122 has been found in HCC, and reactivation of miR-122 can help increase sensitivity of HCC to SOR [14, 15]. So, we believe that miR-122 might be a promising predictor for SOR response in HCC patients.

Taken together, we conducted a retrospective cohort study including hundreds of HBV-related HCC patients with SOR treatment to explore the predictive significance of miR-122 in SOR response and to further reveal the potential effect of the main confounding factor (i.e., HBV load) on such relationship in the patients.

## 2. Materials and Methods

### 2.1. Ethical Principles

The study was approved by the ethics committees of Pingdingshan University and the First People's Hospital of Pingdingshan. All subjects or their families agreed to participate in the study and signed written informed consent forms.

### 2.2. Patients

This study was part of a local HCC research project. All patients who agreed to participate in the project provided their peripheral blood specimens and medical records for scientific research.

Patients with HCC in the First People's Hospital of Pingdingshan between January 2016 and June 2020 were retrospectively included in the study. The included patients should be diagnosed with HBV-related HCC according to American Association for the Study of Liver Diseases criteria [16] and received SOR treatment with a standard dose of 800 mg daily (400 mg, twice a day) for the first time. Before the SOR treatment, the patients should also meet the following criteria: (1) Baseline age ≥ 18 years old. (2) Eastern Cooperative Oncology Group (ECOG) score of 0 or 1 [17]. (3) Child-Pugh classification of A [18]. (4) Absence of hepatitis C, severe fatty liver, or alcoholic liver disease. (5) Presence of macroscopic vascular invasion according to CT scan. (6) Absence of bile duct invasion and distant metastasis according to CT scan and bone scan. (7) Absence of refractory ascites, gastrointestinal hemorrhage, hepatic encephalopathy, or coagulation disorder (INR > 1.5). (8) Absence of other cancers.

**Figure 1:**
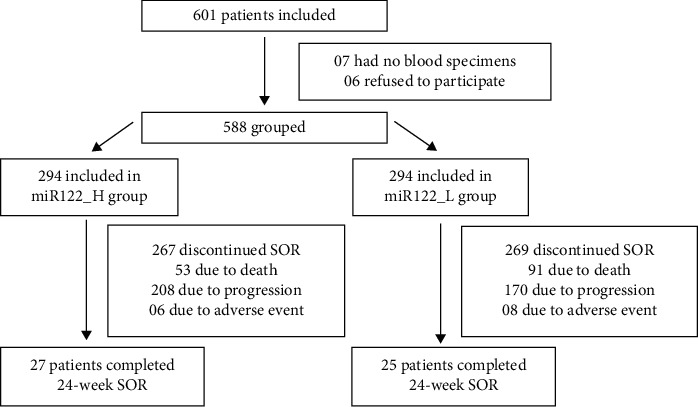
Flow diagram in the study.

A total of 601 HCC patients with SOR treatment completely met the inclusion criteria. Due to no blood specimens and refusal to participate in the study, 13 patients were excluded. So, the remaining 588 patients were included in the study (Figure 1).

In addition, the study enrolled 588 age- and gender-matched healthy controls in 2020 from the Department of Physical Examination in the same hospital. These controls did not have any liver diseases and other confirmed chronic diseases.

### 2.3. Data Collection

Demographic, serological, pathological, and imaging data of the HCC patients were collected from medical records by a group of well-trained investigators. Meanwhile, therapeutic (i.e., adverse event and treatment interruption) and prognostic data (i.e., HCC progression and death) were also collected.

According to the normal range of HBV-DNA in the medical records, HBV-DNA ≥ 10^3^ copies/ml was defined as positive in the study.

### 2.4. Assessment of Efficacy and Outcome

Based on the data from the medical records, the efficacy of SOR and the outcome of the patients during the first 24 weeks after the beginning of the treatment were assessed using several prognostic markers, such as overall response rate, disease control rate, progression-free survival (PFS), and OS.

Progression was confirmed using two standards which were listed below. First, there was no change in the radiological aspect of a lesion according to Response Evaluation Criteria in Solid Tumors (RECIST) criteria [19]. Second, a patient died of HCC or its complications.

RECIST criteria were listed as follows: Complete response (CR) and partial response (PR) were separately defined as the disappearance of all lesions and more than 30% decrease in the sum of all target lesions. Progressive disease (PD) was defined as at least a 20% increase in the sum of all target lesions. Stable disease (SD) was defined as the criteria between PR and PD. The overall response was the sum of CR and PR, and disease control was the sum of CR, PR, and SD.

### 2.5. Measurement of miR-122

As mentioned above, peripheral blood specimen was obtained from each patient when participating in the project. Then, the specimen was centrifugated at 12000 rpm for 10 minutes to separate the serum, and the latter was stored at −70°C for scientific research. In addition, blood specimen was also collected from the controls on admission.

The serum level of miR-122 was measured by RT-qPCR. In the process, U6 snRNA was served as an endogenous control. Forward and reverse primers of miR-122 were 5′-ACACTCCAGCTGGGTGGAGTGTGACAATG-3′ and 5′-CTCAACTGGTGTCG TGGAGTCGGCAATTCAGTTGAGCAAACACC-3′, respectively. Forward and reverse primers of U6 were 5′-CTCGCTTCGGCAGCACA-3′ and 5′-AACGCTTCA CGAATTTGCGT-3′. Brief steps were as follows: First, total RNA was extracted using a ready-to-use TRIzol™ Reagent (Thermo Fisher Scientific). Second, the obtained RNA was transcribed into cDNA using a Hairpin-it™ miRNA or U6 snRNA Normalization Real-time RT-PCR Quantitation Kit (GenePharma). Third, a number of PCR cycles were conducted, and each cycle consisted of three steps: denaturation (95°C for 180 seconds), annealing (62°C for 30 seconds), and elongation (72°C for 30 seconds). Fourth, relative levels of miR-122 in serum were expressed using 2^−△△Ct^ method.

### 2.6. Statistical Analysis

First, the normality of continuous variables in the study was checked by the Shapiro–Wilk test. All the obtained *P* values were larger than 0.05, which indicated that the continuous variables in the study conformed to normal distribution.

Second, the continuous variable in the study was shown as mean and standard deviation, and the difference between the two variables was measured using an independent sample *t*-test. The categorical variable was expressed as frequency and constituent ratio, and the difference between the two variables was determined using the chi-square test. In addition, the difference in PFS or OS between the groups was determined using Kaplan-Meier survival analysis. In these tests, if a *P* value was less than 0.05, it was a statistically significant difference.

Third, the relationship between outcome and serum miR-122 level was measured by multivariate COX regression analysis. Hazard ratio (HR) and 95% confidence interval (CI) were reported. If a 95%CI did not include value one, it was statistically significant. All analysis was conducted using SPSS 17.0.

## 3. Results

### 3.1. Flow Diagram in the Study

In Figure 1, there were 601 HCC patients who met the inclusion criteria. Due to no blood specimens or refusal to join, 13 patients were excluded. The remaining 588 patients were included in the study and were divided into high miR-122 group (miR122_H group) and low miR-122 group (miR122_L group) according to the median of serum miR-122 level at baseline. All of them received SOR, but only 27 patients in the miR122_H group and 25 patients in the miR122_L group completed 24 weeks of treatment. Most of them discontinued SOR treatment due to death, disease progression, or adverse event.

### 3.2. Baseline Level of miR-122 in Serum

**Figure 2:**
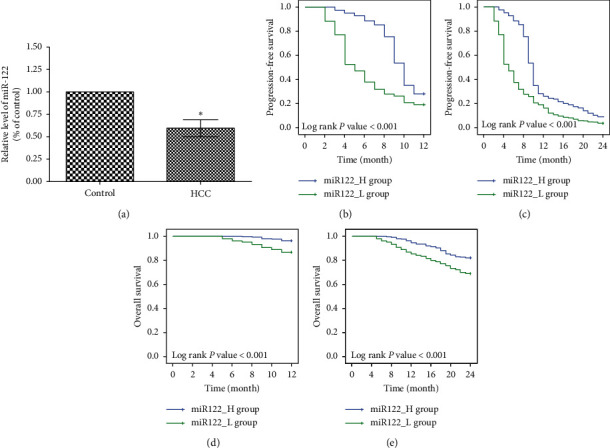
Survival outcomes in the study. (a) Serum level of miR-122 was significantly lower in the HCC patients (*n* = 588) than in the controls (*n* = 588). “*∗*” Indicates *P* < 0.001. (b–e) The patients were divided into miR122_H group (*n* = 294) and miR122_L group (*n* = 294). PFS and OS in the two groups were compared at the 12th week and 24th week using Kaplan–Meier survival analysis.

In Figure 2(a), the serum level of miR-122 was significantly lower in the HCC patients than in the controls (*P* < 0.001). The median of miR-122 in the patients was 0.61, which was the demarcation between the miR122_H and miR122_L groups. Detailed data have been included in Supplementary Table 1.

### 3.3. Characteristics of the Patients in the Study

In Table 1, serum *α*-fetoprotein (AFP) level was higher in the miR122_L group than in the miR122_H group (*P*=0.004). The patients in the miR122_L group had more opportunities to suffer from multiple and bilateral tumors (*P*=0.016and *P*=0.023, respectively). Duration of SOR, overall adverse events, and serious adverse events were equally distributed between the two groups (*P*=0.294, *P*=0.158, and *P*=0.589, respectively).

### 3.4. Radiological Response to SOR in the Study

In Table 2, at the 12th ]week and 24th week, overall response and disease control were more common in the miR122_H group compared with the miR122_L group (12th week: *P*=0.015 and *P*=0.012, respectively; 24th week: *P*=0.019 and *P*=0.007, respectively).

### 3.5. Survival Outcomes in the Study

In Figures 2(b)–2(e), similar to the radiological response, PFS and OS were higher in the miR122_H group than in the miR122_L group both at the 12th week and at the 24th week (12th week: *P* < 0.001 and *P* < 0.001, respectively; 24th week: *P* < 0.001 and *P* < 0.001, respectively).

### 3.6. Prognostic Significance of miR-122 in the Study

In Table 3, multivariate COX regression analyses reported that the higher level of miR-122 at baseline was associated with better overall response, disease control, PFS, and OS at the 12th week (HR: 3.22, 95%CI: 1.19∼8.64; HR: 1.48, 95%CI: 1.08∼1.98; HR: 1.48, 95%CI: 1.08∼1.98; HR: 1.12, 95%CI: 1.07∼1.18). Also, serum level of miR-122 at baseline showed a similar relationship with these radiological and survival markers at the 24th week (HR: 8.02, 95%CI: 1.01∼63.58; HR: 2.47, 95%CI: 1.24∼4.88; HR: 2.47, 95%CI: 1.24∼4.88; HR: 1.20, 95%CI: 1.09∼1.32).

### 3.7. Effect of HBV Load on Predictive Effect of miR-122

In Table 4, the patients were divided into several subgroups according to HBV-DNA or HBeAg status. In the patients with negative HBV-DNA or HBeAg, the higher level of miR-122 at baseline was associated with better 24-week OS (HR: 1.22, 95%CI: 1.09∼1.36; HR: 1.25, 95%CI: 1.13∼1.39).

However, in the patients with positive HBV-DNA or HBeAg, there was no significant relationship between baseline miR-122 level and 24-week OS (HR: 1.12, 95%CI: 0.93∼1.35; HR: 0.99, 95%CI: 0.78∼1.25).

## 4. Discussion

Many HCC patients suffer from macroscopic vascular invasion. It should be an intrahepatic lesion but still indicates a tendency of distant metastasis. Because extrahepatic micrometastasis is difficult to find, single local therapy may not be enough to treat such kind of HCC. So, people have introduced several therapies including SOR. The latter is one oral systemic agent which has been adopted to treat advanced HCC for more than 10 years [20]. It is able to inhibit the development of both intrahepatic lesions and potential metastasis lesions in the body and has achieved satisfactory efficacy.

Nowadays, studies mainly focus on the details of drug usage. For example, Cabibbo et al. report that median OS and median time to radiological progression are separately 10.0 and 4.1 months in the patients with SOR treatment, which help guide doctors to replace second-line therapies in a timely manner [21]. Chen et al. compare SOR with transarterial chemoembolization (TACE) and suggest that dose-adjusted SOR may be cost-effective than TACE for advanced HCC patients, which provide a basis for doctors to improve efficacy and reduce medical costs [22].

Meanwhile, SOR resistance in HCC has always plagued scholars and doctors. Some clinical, pathological, and serological markers have been introduced to predict response to SOR [23–25]. However, they all have some inevitable disadvantages, such as inconvenient measurement and unsatisfactory specificity [23–25]. Therefore, a novel and available predictor is urgently needed for HCC patients.

As one kind of stable molecule in the body, microRNAs exert a variety of biological functions in HCC. However, the prognostic significance of microRNAs in HCC has not been clarified. One study from Yoon et al. has explored several microRNAs including miR-18a, miR-21, miR-139-5p, miR-221, miR-224, and miR-10b-3p in advanced HCC patients but reported that no single microRNA was predictive of response to SOR treatment [26]. In fact, miR-122 is a liver-specific microRNA and its expression in liver tissue far exceeds all the other microRNAs mentioned above [11]. Therefore, the present study focused on serum miR-122 and confirmed the significant relationship between concentration of miR-122 in serum and response to SOR in HCC patients. Specifically, the study revealed that a higher serum level of miR-122 at baseline predicted an about 7-fold and 1.5-fold increase separately in the probabilities of 24-week overall response and disease control under SOR treatment. Similarly, the higher miR-122 level at baseline predicted an about 150% and 20% increase separately in the possibilities of 24-week PFS and OS under SOR treatment.

It is well known that HBV infection is the most important carcinogenic factor for HCC in China [27]. The influence of SOR on host immunity in HCC stratifies by etiology [28], and a high HBV load and antiviral therapy affect the survival of patients treated with SOR [29]. So, it is necessary to explore the effect of HBV infection on prognostic significance of miR-122. In the study, we divided the patients into several subgroups according to HBeAg or HBV-DNA status. The results revealed that a significant relationship between miR-122 and survival outcomes only existed in the patients with negative HBeAg or HBV-DNA. The potential mechanism involved should be explored in future studies.

Many patients in the study were not pathologically diagnosed, which might be a potential limitation. However, HCC is one of the few cancers that can be diagnosed without pathological examination [30]. Furthermore, all the patients were confirmed according to the American Association for the Study of Liver Diseases criteria. So, we did not think that the limitation affected the conclusion.

In addition to SOR, regorafenib is another therapeutic agent that has been demonstrated to be effective in advanced HCC [31]. Recently, some novel immune therapies are under investigation, such as dendritic cell vaccination, immune-modulator strategy, and immune checkpoint inhibition [31]. In order to improve the efficacy, immune therapies are also adopted in conjunction with traditional therapies [32]. However, a unique immune response in the liver favors tolerance, which is a challenge for immune therapies against HCC [31]. So, it is meaningful to explore some effective predictors for response to these therapies mentioned above. Based on the results from the study, we speculate that miR-122 is a potential candidate, which deserves to be explored in future studies.

In conclusion, the study demonstrated that serum miR-122 concentration was associated with radiological and survival outcomes in advanced HCC patients with SOR treatment, and the relatively higher miR-122 level in serum might predict a better response to the drug. Such prognostic significance can be affected by high HBV load or high viral activity. Further studies should be conducted to verify our conclusion.

## Figures and Tables

**Table 1:** Characteristics of the patients in the study.

	miR122_H group^c^ (*n* = 294)	miR122_L group^c^ (*n* = 294)	*P* value
Baseline characteristics			
Male (*n*, %)	254 (86.4)	245 (83.3)	0.300
Age (yrs, mean ± SD)^a^	54.9 ± 12.7	55.4 ± 13.8	0.646
HBeAg positive (*n*, %)	59 (20.1)	68 (23.1)	0.367
HBV-DNA positive (*n*, %)^a,b^	73 (24.8)	77 (26.2)	0.705
Serum ALB (g/L, mean ± SD)^a^	32.9 ± 2.3	33.0 ± 2.4	0.570
Serum TBIL (*μ*mol/L, mean ± SD)^a^	14.2 ± 4.6	13.7 ± 5.0	0.219
Serum AFP (ng/mL, mean ± SD)^a^	302.2 ± 155.3	338.4 ± 152.0	0.004

ECOG score (*n*, %) a			
0 points	119 (40.5)	135 (45.9)	0.183
1 point	175 (59.5)	159 (54.1)	

Maximum diameter of tumor (*n*, %)			
＜5 cm	136 (46.3)	115 (39.1)	0.197
5∼10 cm	146 (49.7)	154 (52.4)	0.062
＞10 cm	12 (4.1)	25 (8.5)	

Number of tumors (*n*, %)			
Single	228 (77.6)	202 (68.7)	0.016
Multiple	66 (22.4)	92 (31.3)	

Extent of tumor (*n*, %)			
Unilateral	263 (89.5)	244 (83.0)	0.023
Bilateral	31 (10.5)	50 (17.0)	

Portal vein invasion (*n*, %)			
Absent	5 (1.7)	6 (2.0)	0.666
Unilateral	191 (65.0)	176 (59.9)	0.214
Bilateral or main	98 (33.3)	112 (38.1)	

Hepatic vein invasion (*n*, %)			
Absent	273 (92.9)	266 (90.5)	0.296
Present	21 (7.1)	28 (9.5)	

SOR treatment			
Duration of SOR (week, mean ± SD)^a^	16.7 ± 4.1	16.3 ± 4.0	0.294
Overall adverse events (*n*, %)	273 (92.9)	281 (95.6)	0.158
Serious adverse events (*n*, %)	6 (2.0)	8 (2.7)	0.589

^a^SD: standard deviation; HBV: hepatitis B virus; ALB: albumin; TBIL: total bilirubin; AFP: *α*-fetoprotein; ECOG: eastern cooperative oncology group; SOR: sorafenib. ^b^Positive HBV-DNA was defined as HBV-DNA ≥ 10^3^ copies/ml. ^c^The patients were divided into the miR122_H group and the miR122_L group according to the median of serum miRNA-122 concentration.

**Table 2:** Radiological response to sorafenib in the study.

Item	miR122_H group (*n* = 294)	miR122_L group (*n* = 294)	*P* value
12-week radiological response			
Complete response (*n*, %)	4	2	―
Partial response (*n*, %)	12	3	―
Stable disease (*n*, %)	67	52	―
Progressive disease (*n*, %)	211	237	―
Intrahepatic (*n*, %)	169	184	―
Extrahepatic (*n*, %)	11	20	―
Both (*n*, %)	31	33	―
Overall response (*n*, %)^a^	16 (5.4)	5 (1.7)	0.015
Disease control (*n*, %)^a^	83 (28.2)	57 (19.4)	0.012

24-week radiological response			
Complete response (*n*, %)	1	0	―
Partial response (*n*, %)	7	1	―
Stable disease (*n*, %)	19	10	―
Progressive disease (*n*, %)	267	283	―
Intrahepatic (*n*, %)	213	225	―
Extrahepatic (*n*, %)	13	17	―
Both (*n*, %)	41	41	―
Overall response (*n*, %)	8 (2.7)	1 (0.3)	0.019
Disease control (*n*, %)	27 (9.2)	11 (3.7)	0.007

^a^“Overall response” was the sum of “complete response” + “partial response,” and “disease control” was the sum of “complete response” + “partial response” + “stable disease.”

**Table 3:** Multivariate association of radiological response and survival outcomes with serum miR-122 concentration.

	Outcomes (*n*)	Total (*n*)	Univariate HR (95%CI)^a^	Multivariate HR (95%CI)^a,b^
12-week overall response				
miR122_L group	5	294	Reference	Reference
miR122_H group	16	294	3.21 (1.19∼8.63)	3.22 (1.19∼8.64)

12-week disease control				
miR122_L group	57	294	Reference	Reference
miR122_H group	83	294	1.47 (1.08∼1.97)	1.48 (1.08∼1.98)

12-week PFS				
miR122_L group	57	294	Reference	Reference
miR122_H group	83	294	1.47 (1.08∼1.97)	1.48 (1.08∼1.98)

12-week OS				
miR122_L group	255	294	Reference	Reference
miR122_H group	283	294	1.12 (1.07∼1.17)	1.12 (1.07∼1.18)

24-week overall response				
miR122_L group	1	294	Reference	Reference
miR122_H group	8	294	8.01 (1.01∼63.57)	8.02 (1.01∼63.58)

24-week disease control				
miR122_L group	11	294	Reference	Reference
miR122_H group	27	294	2.46 (1.24∼4.87)	2.47 (1.24∼4.88)

24-week PFS				
miR122_L group	11	294	Reference	Reference
miR122_H group	27	294	2.46 (1.24∼4.87)	2.47 (1.24∼4.88)

24-week OS				
miR122_L group	203	294	Reference	Reference
miR122_H group	241	294	1.19 (1.09∼1.31)	1.20 (1.09∼1.32)

^a^PFS: progression-free survival; OS: overall survival; HR: hazard ratio; CI: confidence interval. ^b^The multivariate COX model was adjusted by gender, age, annual income, HBeAg, HBV-DNA, serum albumin, serum total bilirubin, serum fetoprotein, ECOG score, maximum diameter of tumor, number of tumors, extent of tumor, portal vein invasion, and hepatic vein invasion.

**Table 4:** Multivariate association of 24-week OS with serum miR-122 concentration according to HBV-DNA or HBeAg status.

	24-week OS^a^ (*n*)	Total (*n*)	Univariate HR (95%CI)^a^	Multivariate HR (95%CI)^a,b^
Positive HBV-DNA^c^				
miR122_L group	54	77	Reference	Reference
miR122_H group	57	73	1.11 (0.92∼1.35)	1.12 (0.93∼1.35)

Negative HBV-DNA				
miR122_L group	149	217	Reference	Reference
miR122_H group	184	221	1.21 (1.09∼1.35)	1.22 (1.09∼1.36)

Positive HBeAg				
miR122_L group	48	68	Reference	Reference
miR122_H group	41	59	0.98 (0.78∼1.24)	0.99 (0.78∼1.25)

Negative HBeAg				
miR122_L group	155	226	Reference	Reference
miR122_H group	200	235	1.24 (1.12∼1.38)	1.25 (1.13∼1.39)

^a^OS: overall survival; HR: hazard ratio; CI: confidence interval. ^b^The multivariate COX model was adjusted by gender, age, annual income, HBeAg, HBV-DNA, serum albumin, serum total bilirubin, serum fetoprotein, ECOG score, maximum diameter of tumor, number of tumors, extent of tumor, portal vein invasion, and hepatic vein invasion. ^c^Positive HBV-DNA was defined as HBV-DNA ≥ 10^3^ copies/ml.

## Data Availability

Data cannot be shared because the data form part of an ongoing study.

## Supplementary Materials

Supplementary Materials Supplementary Table 1: detailed data of miR-122 in RT-qPCR.
