# Supplementary material for: High HBV Load Weakens Predictive Effect of Serum miR-122 on Response to Sorafenib in Hepatocellular Carcinoma Patients
Source: J Oncol. 2021 Jun 11;2021:9938207. doi: 10.1155/2021/9938207 (PMC8214498; doi:10.1155/2021/9938207)
Supplement: Supplementary Materials — Supplementary Table 1: detailed data of miR-122 in RT-qPCR. [file 9938207.f1.doc]

**Supplementary Table 1: detailed data of miR-122 in RT-qPCR.**

|  | HCC | HCC | Control | Control |
| --- | --- | --- | --- | --- |
| No. | Average CT (miR-122) | Average CT (U6) | Average CT (miR-122) | Average CT (U6) |
| 1 | 29.98 | 26.07 | 28.51 | 25.82 |
| 2 | 31.48 | 27.05 | 30.07 | 26.70 |
| 3 | 31.21 | 25.45 | 30.47 | 25.28 |
| 4 | 32.29 | 28.64 | 31.42 | 28.31 |
| 5 | 29.57 | 26.59 | 28.99 | 26.56 |
| 6 | 28.89 | 24.51 | 28.22 | 24.41 |
| 7 | 26.83 | 22.61 | 26.24 | 22.58 |
| 8 | 27.92 | 21.81 | 26.68 | 21.56 |
| 9 | 26.40 | 21.05 | 25.67 | 20.78 |
| 10 | 28.17 | 22.31 | 27.08 | 21.77 |
| 11 | 24.41 | 18.82 | 23.61 | 18.60 |
| 12 | 27.71 | 22.66 | 26.72 | 22.17 |
| 13 | 24.50 | 18.37 | 23.11 | 17.79 |
| 14 | 25.47 | 20.24 | 24.54 | 20.09 |
| 15 | 28.70 | 25.69 | 27.88 | 25.39 |
| 16 | 32.48 | 29.25 | 31.65 | 28.92 |
| 17 | 28.73 | 24.56 | 27.54 | 24.49 |
| 18 | 30.75 | 27.10 | 29.38 | 26.62 |
| 19 | 26.69 | 22.86 | 25.33 | 22.33 |
| 20 | 24.96 | 21.59 | 24.09 | 21.30 |
| 21 | 27.63 | 24.72 | 26.20 | 24.23 |
| 22 | 26.46 | 22.32 | 25.67 | 22.30 |
| 23 | 25.98 | 20.10 | 24.77 | 19.98 |
| 24 | 31.86 | 26.53 | 31.05 | 26.30 |
| 25 | 30.43 | 26.91 | 29.83 | 26.84 |
| 26 | 31.01 | 25.30 | 29.68 | 25.25 |
| 27 | 31.46 | 27.27 | 30.08 | 27.17 |
| 28 | 26.12 | 21.01 | 25.37 | 20.72 |
| 29 | 29.93 | 25.02 | 29.43 | 25.16 |
| 30 | 25.38 | 19.49 | 24.20 | 19.04 |
| 31 | 23.68 | 19.70 | 22.74 | 19.24 |
| 32 | 32.28 | 26.55 | 31.34 | 26.16 |
| 33 | 27.67 | 22.24 | 26.65 | 21.97 |
| 34 | 29.89 | 24.47 | 29.38 | 24.57 |
| 35 | 25.26 | 22.00 | 24.19 | 21.68 |
| 36 | 32.43 | 28.46 | 31.74 | 28.31 |
| 37 | 27.33 | 23.10 | 26.15 | 22.97 |
| 38 | 29.40 | 24.11 | 28.31 | 23.54 |
| 39 | 30.04 | 24.96 | 29.32 | 24.74 |
| 40 | 28.25 | 22.80 | 27.62 | 22.78 |
| 41 | 29.95 | 23.59 | 28.78 | 23.35 |
| 42 | 31.38 | 27.57 | 30.60 | 27.36 |
| 43 | 32.34 | 27.30 | 30.93 | 27.21 |
| 44 | 30.69 | 28.08 | 29.89 | 27.93 |
| 45 | 26.36 | 20.78 | 25.29 | 20.71 |
| 46 | 25.99 | 21.24 | 24.78 | 20.92 |
| 47 | 30.06 | 26.92 | 29.10 | 26.51 |
| 48 | 27.35 | 22.25 | 25.96 | 21.90 |
| 49 | 30.19 | 25.61 | 29.33 | 25.37 |
| 50 | 28.99 | 24.57 | 27.69 | 24.56 |
| 51 | 26.15 | 20.63 | 25.33 | 20.25 |
| 52 | 30.01 | 25.66 | 28.65 | 25.60 |
| 53 | 31.06 | 27.24 | 29.90 | 27.09 |
| 54 | 28.29 | 24.74 | 27.44 | 24.49 |
| 55 | 29.44 | 26.41 | 28.50 | 26.22 |
| 56 | 31.74 | 27.01 | 30.91 | 26.69 |
| 57 | 28.80 | 24.60 | 28.00 | 24.46 |
| 58 | 31.39 | 24.97 | 30.68 | 24.78 |
| 59 | 28.73 | 25.55 | 27.69 | 25.44 |
| 60 | 29.63 | 26.41 | 28.16 | 26.26 |
| 61 | 24.67 | 18.83 | 24.04 | 18.69 |
| 62 | 27.39 | 21.86 | 26.76 | 21.78 |
| 63 | 27.02 | 24.04 | 25.77 | 23.47 |
| 64 | 25.72 | 20.40 | 24.94 | 20.29 |
| 65 | 32.16 | 26.17 | 30.68 | 26.09 |
| 66 | 28.24 | 22.49 | 27.29 | 22.10 |
| 67 | 21.42 | 15.12 | 20.79 | 15.01 |
| 68 | 28.98 | 25.42 | 27.49 | 25.14 |
| 69 | 29.86 | 27.08 | 28.44 | 26.73 |
| 70 | 30.25 | 24.85 | 29.40 | 24.53 |
| 71 | 29.53 | 26.65 | 28.36 | 26.53 |
| 72 | 26.64 | 23.74 | 25.15 | 23.37 |
| 73 | 23.80 | 19.91 | 22.94 | 19.50 |
| 74 | 29.11 | 26.43 | 28.47 | 26.43 |
| 75 | 25.26 | 19.86 | 24.04 | 19.75 |
| 76 | 28.62 | 23.62 | 27.95 | 23.55 |
| 77 | 28.48 | 24.96 | 27.86 | 24.94 |
| 78 | 31.51 | 26.38 | 30.04 | 26.10 |
| 79 | 31.82 | 26.81 | 31.10 | 26.61 |
| 80 | 25.76 | 19.62 | 25.12 | 19.42 |
| 81 | 28.25 | 25.41 | 27.38 | 25.28 |
| 82 | 27.45 | 24.18 | 26.69 | 23.97 |
| 83 | 27.81 | 22.59 | 27.02 | 22.31 |
| 84 | 28.31 | 22.59 | 27.57 | 22.43 |
| 85 | 30.05 | 23.86 | 29.31 | 23.73 |
| 86 | 28.73 | 22.80 | 27.71 | 22.63 |
| 87 | 29.22 | 25.36 | 28.09 | 24.93 |
| 88 | 30.33 | 27.62 | 29.59 | 27.39 |
| 89 | 29.80 | 27.10 | 28.55 | 27.01 |
| 90 | 31.53 | 28.12 | 30.86 | 27.98 |
| 91 | 30.24 | 27.31 | 29.18 | 26.77 |
| 92 | 27.88 | 23.20 | 27.24 | 23.16 |
| 93 | 30.25 | 25.86 | 29.26 | 25.47 |
| 94 | 27.32 | 23.76 | 25.98 | 23.41 |
| 95 | 28.70 | 25.41 | 28.08 | 25.30 |
| 96 | 26.21 | 20.78 | 25.80 | 20.84 |
| 97 | 30.59 | 24.55 | 29.81 | 24.27 |
| 98 | 28.80 | 26.24 | 27.93 | 25.91 |
| 99 | 28.97 | 25.64 | 27.47 | 25.15 |
| 100 | 27.38 | 21.80 | 25.97 | 21.51 |
| 101 | 25.63 | 20.17 | 24.27 | 20.15 |
| 102 | 30.20 | 23.75 | 29.28 | 23.66 |
| 103 | 25.78 | 22.45 | 24.65 | 22.05 |
| 104 | 32.27 | 29.69 | 31.03 | 29.29 |
| 105 | 28.14 | 22.26 | 26.81 | 22.02 |
| 106 | 26.11 | 23.41 | 25.12 | 22.99 |
| 107 | 27.07 | 23.36 | 25.96 | 23.08 |
| 108 | 31.57 | 25.30 | 30.63 | 25.27 |
| 109 | 31.13 | 25.24 | 30.40 | 25.07 |
| 110 | 30.13 | 26.78 | 29.48 | 26.78 |
| 111 | 27.46 | 24.46 | 25.98 | 23.91 |
| 112 | 24.89 | 19.96 | 24.34 | 19.89 |
| 113 | 24.20 | 17.97 | 23.69 | 17.95 |
| 114 | 26.11 | 20.11 | 25.40 | 19.93 |
| 115 | 28.20 | 23.27 | 26.93 | 22.99 |
| 116 | 25.94 | 21.92 | 24.67 | 21.57 |
| 117 | 28.09 | 24.28 | 26.94 | 24.03 |
| 118 | 26.70 | 22.42 | 26.02 | 22.37 |
| 119 | 25.35 | 21.43 | 24.47 | 21.19 |
| 120 | 30.73 | 26.14 | 29.86 | 25.89 |
| 121 | 29.25 | 26.19 | 28.18 | 25.63 |
| 122 | 30.27 | 23.96 | 29.57 | 23.86 |
| 123 | 26.30 | 23.60 | 25.09 | 23.25 |
| 124 | 31.96 | 26.86 | 30.78 | 26.79 |
| 125 | 26.10 | 22.43 | 25.02 | 22.15 |
| 126 | 27.36 | 21.31 | 26.68 | 21.15 |
| 127 | 32.10 | 25.96 | 31.19 | 25.71 |
| 128 | 27.43 | 22.86 | 26.92 | 22.91 |
| 129 | 26.16 | 19.96 | 24.89 | 19.42 |
| 130 | 26.28 | 22.96 | 25.68 | 22.90 |
| 131 | 30.93 | 27.34 | 30.37 | 27.28 |
| 132 | 27.31 | 23.53 | 26.49 | 23.46 |
| 133 | 28.81 | 24.80 | 27.62 | 24.63 |
| 134 | 25.55 | 22.53 | 24.77 | 22.25 |
| 135 | 29.70 | 26.47 | 28.52 | 25.88 |
| 136 | 24.78 | 19.46 | 23.82 | 18.92 |
| 137 | 28.81 | 26.26 | 28.21 | 26.34 |
| 138 | 23.71 | 20.73 | 22.70 | 20.20 |
| 139 | 23.66 | 18.29 | 22.32 | 18.24 |
| 140 | 30.95 | 27.60 | 29.95 | 27.22 |
| 141 | 29.12 | 25.34 | 28.56 | 25.34 |
| 142 | 30.12 | 23.73 | 29.15 | 23.62 |
| 143 | 24.57 | 19.75 | 23.81 | 19.40 |
| 144 | 25.46 | 20.67 | 24.66 | 20.65 |
| 145 | 28.32 | 22.27 | 27.32 | 22.10 |
| 146 | 30.42 | 24.89 | 29.13 | 24.42 |
| 147 | 26.39 | 22.64 | 25.58 | 22.34 |
| 148 | 30.08 | 27.02 | 29.12 | 26.88 |
| 149 | 30.74 | 25.44 | 29.36 | 25.33 |
| 150 | 24.69 | 20.89 | 23.33 | 20.58 |
| 151 | 24.51 | 19.78 | 23.17 | 19.53 |
| 152 | 25.96 | 23.21 | 25.44 | 23.25 |
| 153 | 25.94 | 22.69 | 24.45 | 22.21 |
| 154 | 31.06 | 28.19 | 29.85 | 27.71 |
| 155 | 30.34 | 26.64 | 29.39 | 26.19 |
| 156 | 26.49 | 20.95 | 25.38 | 20.62 |
| 157 | 29.15 | 24.32 | 28.47 | 24.15 |
| 158 | 27.36 | 21.27 | 26.37 | 20.81 |
| 159 | 29.99 | 25.75 | 28.94 | 25.66 |
| 160 | 31.31 | 27.14 | 30.53 | 26.88 |
| 161 | 29.05 | 25.23 | 28.22 | 25.05 |
| 162 | 28.00 | 23.54 | 26.58 | 23.30 |
| 163 | 30.29 | 26.09 | 29.10 | 25.71 |
| 164 | 31.26 | 25.93 | 30.35 | 25.88 |
| 165 | 26.73 | 20.62 | 26.03 | 20.39 |
| 166 | 25.67 | 19.57 | 25.25 | 19.56 |
| 167 | 28.33 | 23.53 | 27.35 | 23.39 |
| 168 | 28.36 | 23.23 | 27.13 | 22.83 |
| 169 | 26.78 | 21.67 | 25.60 | 21.12 |
| 170 | 31.98 | 26.01 | 30.94 | 25.56 |
| 171 | 30.44 | 24.52 | 29.68 | 24.39 |
| 172 | 31.48 | 25.91 | 30.02 | 25.52 |
| 173 | 31.88 | 26.56 | 30.58 | 26.03 |
| 174 | 31.20 | 27.26 | 29.92 | 27.04 |
| 175 | 25.15 | 22.34 | 24.11 | 21.83 |
| 176 | 28.48 | 24.76 | 27.11 | 24.55 |
| 177 | 26.33 | 21.01 | 25.47 | 21.00 |
| 178 | 29.12 | 25.13 | 28.42 | 24.97 |
| 179 | 27.81 | 21.72 | 26.34 | 21.30 |
| 180 | 29.22 | 24.99 | 28.02 | 24.70 |
| 181 | 25.68 | 19.76 | 24.65 | 19.17 |
| 182 | 27.05 | 21.00 | 25.73 | 20.48 |
| 183 | 29.98 | 24.93 | 29.04 | 24.56 |
| 184 | 29.28 | 23.08 | 28.62 | 23.03 |
| 185 | 29.58 | 25.15 | 28.61 | 24.76 |
| 186 | 24.23 | 20.62 | 22.95 | 20.15 |
| 187 | 30.18 | 27.62 | 28.91 | 27.20 |
| 188 | 30.36 | 27.27 | 29.11 | 26.84 |
| 189 | 25.66 | 19.48 | 24.86 | 19.31 |
| 190 | 32.25 | 27.25 | 30.86 | 26.98 |
| 191 | 26.90 | 22.96 | 25.74 | 22.42 |
| 192 | 27.60 | 23.75 | 27.03 | 23.68 |
| 193 | 26.59 | 22.38 | 25.94 | 22.25 |
| 194 | 29.31 | 25.80 | 28.42 | 25.79 |
| 195 | 29.20 | 24.50 | 28.20 | 24.15 |
| 196 | 30.82 | 27.98 | 29.40 | 27.47 |
| 197 | 25.72 | 20.90 | 25.15 | 20.81 |
| 198 | 32.98 | 28.83 | 32.38 | 28.77 |
| 199 | 32.31 | 28.44 | 31.11 | 28.32 |
| 200 | 27.98 | 25.34 | 27.45 | 25.32 |
| 201 | 32.36 | 26.32 | 31.26 | 26.02 |
| 202 | 27.45 | 21.91 | 26.49 | 21.85 |
| 203 | 30.79 | 26.32 | 29.72 | 25.91 |
| 204 | 28.81 | 25.69 | 27.78 | 25.42 |
| 205 | 32.25 | 27.76 | 31.54 | 27.68 |
| 206 | 29.81 | 27.24 | 28.49 | 26.93 |
| 207 | 26.70 | 23.62 | 25.32 | 23.33 |
| 208 | 25.36 | 22.02 | 23.88 | 21.58 |
| 209 | 23.63 | 19.82 | 22.96 | 19.80 |
| 210 | 23.74 | 19.45 | 22.73 | 19.40 |
| 211 | 28.93 | 24.05 | 27.86 | 23.53 |
| 212 | 31.05 | 28.17 | 29.66 | 28.08 |
| 213 | 28.79 | 22.52 | 27.60 | 22.51 |
| 214 | 29.89 | 26.85 | 28.80 | 26.79 |
| 215 | 28.40 | 23.84 | 27.79 | 23.74 |
| 216 | 30.19 | 27.26 | 29.29 | 27.22 |
| 217 | 28.40 | 22.68 | 27.54 | 22.34 |
| 218 | 28.15 | 25.21 | 27.13 | 25.21 |
| 219 | 26.80 | 23.51 | 25.32 | 23.30 |
| 220 | 29.83 | 23.59 | 29.19 | 23.64 |
| 221 | 25.85 | 22.72 | 24.46 | 22.67 |
| 222 | 27.81 | 21.90 | 26.98 | 21.67 |
| 223 | 28.14 | 24.39 | 27.22 | 24.11 |
| 224 | 29.46 | 23.22 | 28.76 | 23.03 |
| 225 | 24.70 | 18.71 | 23.39 | 18.20 |
| 226 | 31.88 | 26.19 | 31.08 | 25.90 |
| 227 | 26.71 | 22.45 | 25.98 | 22.27 |
| 228 | 23.61 | 17.95 | 22.24 | 17.71 |
| 229 | 31.44 | 27.28 | 30.52 | 27.22 |
| 230 | 23.58 | 19.11 | 22.39 | 18.87 |
| 231 | 27.52 | 22.50 | 26.72 | 22.43 |
| 232 | 31.91 | 27.46 | 30.47 | 26.90 |
| 233 | 24.58 | 20.94 | 23.56 | 20.68 |
| 234 | 28.17 | 23.77 | 27.10 | 23.28 |
| 235 | 29.26 | 22.90 | 28.36 | 22.52 |
| 236 | 29.32 | 26.35 | 28.49 | 26.33 |
| 237 | 28.12 | 23.52 | 27.40 | 23.37 |
| 238 | 27.63 | 24.55 | 26.48 | 24.47 |
| 239 | 28.68 | 25.40 | 27.68 | 25.31 |
| 240 | 28.35 | 25.28 | 26.89 | 24.94 |
| 241 | 28.43 | 25.13 | 27.06 | 24.73 |
| 242 | 26.83 | 22.08 | 25.69 | 21.85 |
| 243 | 32.02 | 26.11 | 30.86 | 25.64 |
| 244 | 25.30 | 22.64 | 24.07 | 22.53 |
| 245 | 27.31 | 23.64 | 26.07 | 23.48 |
| 246 | 28.93 | 24.52 | 28.30 | 24.46 |
| 247 | 24.85 | 21.94 | 23.85 | 21.91 |
| 248 | 25.49 | 20.50 | 24.33 | 20.08 |
| 249 | 31.81 | 27.46 | 30.77 | 27.00 |
| 250 | 24.91 | 21.72 | 24.06 | 21.67 |
| 251 | 34.35 | 28.27 | 33.01 | 28.08 |
| 252 | 31.47 | 25.83 | 30.82 | 25.71 |
| 253 | 27.17 | 23.16 | 25.67 | 22.96 |
| 254 | 28.45 | 25.79 | 27.49 | 25.69 |
| 255 | 26.40 | 21.32 | 25.50 | 20.98 |
| 256 | 26.65 | 21.02 | 25.50 | 20.79 |
| 257 | 26.24 | 22.96 | 25.44 | 22.67 |
| 258 | 24.35 | 21.16 | 23.71 | 20.97 |
| 259 | 25.72 | 20.13 | 24.66 | 19.76 |
| 260 | 29.22 | 25.28 | 28.34 | 24.98 |
| 261 | 28.34 | 22.05 | 27.44 | 21.85 |
| 262 | 28.87 | 23.25 | 28.21 | 23.10 |
| 263 | 22.24 | 17.85 | 21.32 | 17.41 |
| 264 | 26.57 | 23.16 | 26.04 | 23.19 |
| 265 | 26.35 | 21.78 | 25.14 | 21.30 |
| 266 | 27.93 | 21.84 | 26.62 | 21.65 |
| 267 | 32.49 | 28.68 | 31.19 | 28.58 |
| 268 | 28.65 | 24.39 | 27.97 | 24.29 |
| 269 | 29.39 | 24.20 | 27.99 | 23.60 |
| 270 | 26.96 | 23.22 | 25.69 | 23.12 |
| 271 | 30.47 | 27.75 | 29.19 | 27.18 |
| 272 | 28.81 | 23.22 | 27.44 | 22.68 |
| 273 | 24.14 | 17.80 | 23.40 | 17.96 |
| 274 | 32.20 | 26.78 | 30.94 | 26.30 |
| 275 | 25.06 | 20.75 | 24.65 | 20.77 |
| 276 | 29.19 | 23.20 | 27.76 | 22.71 |
| 277 | 28.04 | 24.03 | 26.56 | 23.45 |
| 278 | 28.70 | 23.33 | 28.20 | 23.39 |
| 279 | 31.73 | 27.23 | 30.81 | 26.92 |
| 280 | 27.25 | 23.06 | 26.21 | 22.86 |
| 281 | 26.09 | 20.33 | 25.05 | 20.04 |
| 282 | 24.22 | 18.23 | 23.34 | 18.22 |
| 283 | 27.51 | 21.79 | 26.93 | 21.81 |
| 284 | 28.05 | 24.24 | 27.35 | 24.19 |
| 285 | 30.65 | 26.26 | 29.87 | 26.06 |
| 286 | 24.06 | 19.17 | 23.62 | 19.16 |
| 287 | 27.34 | 21.79 | 26.22 | 21.24 |
| 288 | 31.11 | 27.52 | 29.95 | 26.98 |
| 289 | 25.94 | 20.88 | 24.70 | 20.70 |
| 290 | 24.74 | 19.87 | 23.53 | 19.35 |
| 291 | 29.17 | 26.16 | 28.49 | 25.99 |
| 292 | 24.88 | 20.72 | 24.27 | 20.53 |
| 293 | 27.35 | 22.98 | 26.20 | 22.64 |
| 294 | 25.40 | 21.30 | 23.99 | 21.27 |
| 295 | 26.26 | 20.13 | 25.36 | 19.95 |
| 296 | 24.46 | 18.26 | 23.98 | 18.25 |
| 297 | 25.63 | 21.30 | 24.65 | 21.23 |
| 298 | 28.16 | 24.43 | 27.35 | 24.37 |
| 299 | 33.49 | 28.08 | 32.52 | 27.69 |
| 300 | 27.78 | 22.11 | 27.04 | 22.10 |
| 301 | 32.13 | 28.05 | 30.84 | 27.48 |
| 302 | 25.64 | 20.26 | 24.59 | 19.99 |
| 303 | 29.10 | 24.57 | 28.18 | 24.21 |
| 304 | 28.45 | 22.36 | 27.69 | 22.15 |
| 305 | 30.51 | 26.84 | 29.27 | 26.25 |
| 306 | 27.76 | 21.34 | 27.08 | 21.31 |
| 307 | 27.05 | 24.45 | 26.43 | 24.42 |
| 308 | 32.09 | 27.84 | 30.68 | 27.60 |
| 309 | 25.96 | 19.86 | 25.12 | 19.64 |
| 310 | 29.65 | 25.85 | 28.46 | 25.48 |
| 311 | 27.68 | 22.89 | 26.79 | 22.87 |
| 312 | 29.35 | 25.55 | 28.38 | 25.12 |
| 313 | 28.38 | 24.55 | 27.13 | 24.11 |
| 314 | 29.17 | 23.41 | 27.87 | 22.92 |
| 315 | 26.69 | 23.77 | 26.18 | 23.78 |
| 316 | 27.81 | 22.35 | 26.88 | 21.93 |
| 317 | 29.04 | 24.81 | 27.85 | 24.42 |
| 318 | 25.48 | 22.72 | 24.66 | 22.60 |
| 319 | 25.55 | 21.75 | 24.94 | 21.69 |
| 320 | 25.62 | 21.69 | 24.17 | 21.34 |
| 321 | 32.12 | 29.15 | 31.51 | 29.11 |
| 322 | 24.76 | 18.65 | 23.98 | 18.64 |
| 323 | 30.79 | 27.57 | 30.02 | 27.31 |
| 324 | 25.89 | 20.67 | 24.73 | 20.60 |
| 325 | 24.87 | 19.91 | 23.93 | 19.53 |
| 326 | 28.53 | 23.65 | 27.82 | 23.61 |
| 327 | 25.91 | 21.34 | 24.82 | 21.15 |
| 328 | 29.30 | 25.47 | 28.14 | 25.14 |
| 329 | 29.09 | 24.72 | 27.75 | 24.46 |
| 330 | 27.66 | 24.85 | 26.20 | 24.47 |
| 331 | 32.05 | 25.98 | 30.88 | 25.93 |
| 332 | 28.58 | 25.62 | 27.32 | 25.51 |
| 333 | 23.56 | 20.29 | 22.77 | 20.17 |
| 334 | 31.19 | 27.28 | 30.42 | 27.12 |
| 335 | 29.83 | 24.00 | 28.74 | 23.45 |
| 336 | 31.18 | 24.89 | 30.57 | 24.84 |
| 337 | 29.73 | 26.66 | 28.38 | 26.13 |
| 338 | 27.20 | 23.24 | 26.27 | 23.14 |
| 339 | 31.88 | 28.10 | 30.43 | 27.58 |
| 340 | 23.84 | 18.48 | 22.92 | 18.35 |
| 341 | 28.69 | 22.94 | 27.70 | 22.52 |
| 342 | 30.61 | 25.92 | 29.72 | 25.54 |
| 343 | 24.78 | 18.99 | 23.77 | 18.49 |
| 344 | 28.94 | 24.17 | 28.10 | 23.91 |
| 345 | 28.65 | 25.29 | 27.86 | 25.09 |
| 346 | 29.90 | 24.36 | 28.99 | 24.34 |
| 347 | 27.41 | 24.90 | 25.97 | 24.39 |
| 348 | 31.87 | 28.01 | 31.25 | 27.96 |
| 349 | 23.75 | 17.67 | 22.93 | 17.48 |
| 350 | 25.04 | 19.67 | 23.89 | 19.43 |
| 351 | 27.38 | 22.46 | 26.01 | 22.08 |
| 352 | 32.36 | 28.78 | 31.45 | 28.61 |
| 353 | 27.22 | 22.68 | 25.94 | 22.23 |
| 354 | 28.74 | 24.14 | 27.47 | 23.58 |
| 355 | 27.48 | 24.67 | 26.68 | 24.40 |
| 356 | 31.13 | 28.20 | 29.66 | 27.65 |
| 357 | 28.24 | 24.58 | 26.94 | 24.18 |
| 358 | 29.93 | 25.01 | 29.12 | 24.74 |
| 359 | 26.22 | 23.64 | 25.40 | 23.36 |
| 360 | 31.06 | 26.50 | 29.99 | 26.33 |
| 361 | 30.34 | 24.42 | 29.81 | 24.43 |
| 362 | 25.03 | 20.41 | 24.32 | 20.17 |
| 363 | 24.95 | 21.42 | 24.29 | 21.27 |
| 364 | 23.48 | 20.68 | 22.84 | 20.59 |
| 365 | 29.92 | 26.36 | 28.50 | 26.18 |
| 366 | 31.34 | 27.70 | 30.71 | 27.63 |
| 367 | 29.86 | 24.33 | 28.60 | 23.75 |
| 368 | 27.24 | 24.67 | 26.18 | 24.47 |
| 369 | 32.00 | 27.74 | 30.71 | 27.44 |
| 370 | 26.71 | 23.51 | 25.75 | 23.14 |
| 371 | 26.18 | 22.07 | 24.90 | 21.62 |
| 372 | 30.69 | 26.71 | 29.94 | 26.55 |
| 373 | 26.95 | 23.64 | 26.26 | 23.55 |
| 374 | 29.64 | 23.80 | 28.37 | 23.52 |
| 375 | 24.84 | 21.47 | 24.03 | 21.25 |
| 376 | 28.97 | 24.59 | 28.43 | 24.74 |
| 377 | 27.27 | 24.22 | 26.02 | 23.98 |
| 378 | 28.47 | 24.95 | 27.80 | 24.86 |
| 379 | 26.98 | 23.44 | 25.83 | 23.02 |
| 380 | 30.18 | 25.37 | 29.07 | 25.27 |
| 381 | 29.59 | 26.05 | 28.10 | 25.93 |
| 382 | 31.41 | 27.66 | 30.88 | 27.80 |
| 383 | 31.06 | 28.24 | 29.64 | 28.23 |
| 384 | 27.53 | 24.80 | 26.43 | 24.50 |
| 385 | 30.91 | 26.69 | 29.99 | 26.56 |
| 386 | 28.39 | 22.08 | 27.44 | 21.67 |
| 387 | 30.85 | 27.27 | 30.27 | 27.24 |
| 388 | 28.44 | 22.48 | 27.07 | 21.89 |
| 389 | 27.83 | 23.58 | 27.02 | 23.31 |
| 390 | 26.54 | 23.81 | 25.88 | 23.66 |
| 391 | 31.80 | 28.25 | 30.34 | 28.15 |
| 392 | 27.83 | 21.95 | 27.33 | 21.95 |
| 393 | 28.62 | 22.95 | 27.52 | 22.47 |
| 394 | 26.68 | 21.70 | 26.12 | 21.73 |
| 395 | 28.82 | 22.87 | 27.86 | 22.58 |
| 396 | 27.92 | 24.85 | 26.44 | 24.64 |
| 397 | 30.89 | 24.74 | 30.15 | 24.76 |
| 398 | 30.59 | 24.26 | 29.51 | 24.20 |
| 399 | 30.56 | 27.40 | 29.40 | 27.19 |
| 400 | 28.50 | 25.25 | 27.57 | 24.87 |
| 401 | 31.65 | 29.09 | 30.97 | 28.91 |
| 402 | 30.95 | 24.73 | 30.39 | 24.74 |
| 403 | 29.08 | 23.60 | 27.64 | 23.47 |
| 404 | 27.72 | 25.14 | 27.09 | 25.01 |
| 405 | 26.01 | 21.57 | 25.25 | 21.37 |
| 406 | 28.97 | 23.13 | 28.41 | 23.24 |
| 407 | 31.69 | 27.05 | 30.92 | 26.83 |
| 408 | 23.78 | 20.89 | 22.91 | 20.80 |
| 409 | 26.15 | 23.30 | 24.98 | 22.94 |
| 410 | 29.66 | 24.96 | 28.79 | 24.60 |
| 411 | 25.37 | 22.43 | 24.65 | 22.25 |
| 412 | 25.10 | 22.28 | 23.98 | 21.95 |
| 413 | 31.37 | 26.56 | 30.83 | 26.53 |
| 414 | 29.49 | 26.66 | 28.05 | 26.17 |
| 415 | 27.17 | 21.94 | 26.48 | 21.76 |
| 416 | 29.17 | 23.79 | 27.86 | 23.56 |
| 417 | 29.17 | 23.95 | 27.76 | 23.46 |
| 418 | 27.72 | 22.46 | 26.25 | 21.97 |
| 419 | 29.07 | 24.18 | 27.67 | 23.63 |
| 420 | 29.55 | 26.78 | 28.29 | 26.59 |
| 421 | 29.18 | 23.31 | 27.70 | 23.26 |
| 422 | 28.46 | 23.50 | 27.86 | 23.47 |
| 423 | 31.84 | 25.86 | 31.06 | 25.67 |
| 424 | 30.26 | 24.52 | 29.37 | 24.46 |
| 425 | 29.99 | 24.99 | 29.12 | 24.76 |
| 426 | 30.70 | 27.26 | 29.24 | 26.81 |
| 427 | 28.18 | 24.96 | 27.12 | 24.58 |
| 428 | 28.00 | 23.15 | 26.73 | 22.76 |
| 429 | 28.70 | 23.12 | 27.67 | 22.74 |
| 430 | 27.94 | 22.50 | 27.28 | 22.42 |
| 431 | 26.68 | 20.35 | 25.42 | 19.86 |
| 432 | 28.12 | 24.10 | 26.93 | 23.94 |
| 433 | 31.23 | 25.41 | 30.35 | 25.12 |
| 434 | 30.06 | 25.51 | 28.92 | 25.14 |
| 435 | 29.86 | 25.68 | 28.38 | 25.43 |
| 436 | 31.38 | 28.86 | 30.54 | 28.55 |
| 437 | 28.89 | 24.99 | 28.30 | 24.94 |
| 438 | 31.80 | 28.16 | 30.52 | 28.12 |
| 439 | 25.08 | 22.48 | 23.72 | 22.32 |
| 440 | 28.48 | 24.61 | 27.01 | 24.03 |
| 441 | 27.88 | 23.64 | 26.77 | 23.52 |
| 442 | 28.22 | 25.04 | 27.39 | 24.83 |
| 443 | 26.40 | 22.71 | 25.61 | 22.51 |
| 444 | 29.66 | 24.70 | 28.96 | 24.63 |
| 445 | 28.09 | 23.25 | 27.03 | 23.13 |
| 446 | 27.37 | 24.23 | 26.54 | 24.00 |
| 447 | 28.61 | 25.59 | 27.54 | 25.06 |
| 448 | 30.53 | 25.50 | 29.96 | 25.46 |
| 449 | 26.08 | 21.26 | 25.13 | 20.84 |
| 450 | 26.67 | 20.68 | 26.14 | 20.67 |
| 451 | 31.26 | 28.74 | 30.27 | 28.58 |
| 452 | 31.64 | 25.86 | 30.64 | 25.77 |
| 453 | 24.86 | 21.42 | 24.25 | 21.34 |
| 454 | 31.28 | 28.25 | 29.99 | 27.78 |
| 455 | 29.48 | 24.27 | 28.73 | 24.12 |
| 456 | 26.98 | 24.14 | 26.15 | 23.89 |
| 457 | 30.77 | 24.75 | 29.66 | 24.61 |
| 458 | 26.50 | 22.22 | 25.05 | 22.21 |
| 459 | 29.45 | 25.85 | 28.86 | 25.82 |
| 460 | 24.32 | 19.32 | 23.09 | 19.02 |
| 461 | 30.12 | 23.86 | 28.73 | 23.58 |
| 462 | 25.64 | 21.86 | 24.37 | 21.79 |
| 463 | 29.96 | 25.74 | 28.68 | 25.53 |
| 464 | 29.96 | 26.34 | 28.65 | 25.97 |
| 465 | 25.68 | 22.09 | 24.84 | 22.06 |
| 466 | 24.47 | 20.43 | 23.22 | 20.15 |
| 467 | 27.18 | 22.72 | 26.45 | 22.62 |
| 468 | 30.88 | 24.83 | 29.92 | 24.74 |
| 469 | 26.56 | 21.48 | 25.18 | 20.93 |
| 470 | 31.70 | 26.41 | 31.01 | 26.27 |
| 471 | 26.65 | 22.71 | 25.28 | 22.24 |
| 472 | 31.18 | 25.98 | 30.27 | 25.93 |
| 473 | 26.65 | 23.59 | 26.11 | 23.65 |
| 474 | 31.43 | 25.51 | 30.23 | 25.08 |
| 475 | 26.42 | 20.13 | 25.46 | 19.58 |
| 476 | 29.76 | 24.11 | 28.70 | 23.75 |
| 477 | 30.21 | 27.06 | 29.28 | 26.98 |
| 478 | 27.16 | 24.41 | 26.57 | 24.42 |
| 479 | 24.09 | 19.34 | 22.77 | 19.05 |
| 480 | 27.37 | 22.46 | 26.16 | 22.17 |
| 481 | 30.35 | 24.54 | 29.34 | 24.05 |
| 482 | 28.12 | 23.91 | 26.74 | 23.77 |
| 483 | 24.52 | 19.64 | 23.77 | 19.35 |
| 484 | 31.30 | 28.44 | 30.75 | 28.42 |
| 485 | 28.44 | 24.69 | 27.59 | 24.56 |
| 486 | 30.07 | 26.32 | 29.55 | 26.31 |
| 487 | 30.17 | 27.66 | 29.12 | 27.33 |
| 488 | 24.72 | 18.71 | 23.95 | 18.35 |
| 489 | 30.88 | 26.09 | 29.42 | 26.03 |
| 490 | 27.74 | 22.19 | 27.21 | 22.24 |
| 491 | 29.18 | 25.63 | 28.03 | 25.55 |
| 492 | 28.25 | 24.04 | 26.80 | 23.70 |
| 493 | 28.16 | 23.27 | 27.59 | 23.42 |
| 494 | 24.57 | 18.71 | 23.45 | 18.30 |
| 495 | 24.01 | 18.49 | 22.71 | 18.03 |
| 496 | 26.01 | 23.02 | 25.02 | 22.58 |
| 497 | 24.38 | 18.07 | 23.55 | 17.80 |
| 498 | 32.06 | 27.73 | 31.40 | 27.61 |
| 499 | 30.57 | 26.05 | 29.62 | 26.04 |
| 500 | 32.11 | 28.27 | 31.57 | 28.31 |
| 501 | 28.55 | 24.81 | 27.83 | 24.76 |
| 502 | 25.09 | 19.07 | 23.83 | 18.82 |
| 503 | 24.14 | 21.27 | 22.79 | 20.85 |
| 504 | 30.89 | 27.43 | 29.50 | 26.84 |
| 505 | 25.68 | 20.29 | 24.56 | 19.80 |
| 506 | 26.70 | 21.38 | 25.98 | 21.22 |
| 507 | 24.67 | 19.06 | 23.98 | 18.93 |
| 508 | 28.85 | 22.73 | 27.85 | 22.46 |
| 509 | 29.56 | 23.19 | 28.95 | 23.10 |
| 510 | 26.42 | 21.08 | 25.34 | 21.07 |
| 511 | 30.06 | 26.40 | 29.08 | 25.97 |
| 512 | 27.51 | 22.38 | 26.60 | 22.01 |
| 513 | 31.68 | 27.06 | 30.98 | 26.90 |
| 514 | 23.87 | 19.60 | 22.89 | 19.51 |
| 515 | 29.86 | 26.54 | 28.60 | 26.03 |
| 516 | 30.78 | 25.46 | 30.22 | 25.45 |
| 517 | 30.19 | 26.71 | 28.95 | 26.16 |
| 518 | 29.81 | 23.64 | 29.20 | 23.56 |
| 519 | 33.17 | 29.99 | 31.91 | 29.65 |
| 520 | 29.40 | 24.67 | 28.11 | 24.28 |
| 521 | 28.73 | 23.66 | 27.48 | 23.16 |
| 522 | 28.39 | 24.86 | 27.59 | 24.63 |
| 523 | 30.12 | 27.05 | 29.58 | 27.03 |
| 524 | 31.02 | 25.10 | 30.10 | 25.00 |
| 525 | 29.86 | 26.53 | 28.45 | 26.10 |
| 526 | 32.18 | 27.47 | 31.04 | 27.25 |
| 527 | 30.65 | 24.20 | 29.19 | 24.07 |
| 528 | 26.30 | 23.53 | 24.93 | 23.34 |
| 529 | 31.94 | 25.55 | 30.86 | 25.38 |
| 530 | 31.00 | 26.08 | 29.58 | 25.92 |
| 531 | 28.09 | 24.46 | 27.49 | 24.39 |
| 532 | 29.08 | 25.86 | 27.64 | 25.52 |
| 533 | 31.81 | 26.45 | 30.52 | 26.19 |
| 534 | 26.42 | 23.07 | 25.08 | 22.86 |
| 535 | 27.26 | 23.08 | 26.74 | 23.10 |
| 536 | 26.71 | 24.11 | 25.87 | 24.08 |
| 537 | 27.02 | 24.05 | 25.57 | 23.75 |
| 538 | 27.07 | 21.31 | 25.85 | 21.12 |
| 539 | 29.96 | 26.20 | 28.88 | 25.82 |
| 540 | 25.86 | 21.93 | 25.03 | 21.69 |
| 541 | 28.99 | 22.52 | 27.96 | 22.42 |
| 542 | 30.70 | 27.00 | 29.29 | 26.92 |
| 543 | 26.71 | 23.62 | 26.15 | 23.56 |
| 544 | 31.80 | 28.84 | 30.87 | 28.71 |
| 545 | 30.15 | 25.95 | 28.87 | 25.85 |
| 546 | 32.47 | 29.46 | 31.70 | 29.28 |
| 547 | 30.89 | 24.89 | 29.44 | 24.74 |
| 548 | 29.12 | 25.63 | 28.09 | 25.26 |
| 549 | 27.54 | 21.33 | 26.70 | 21.00 |
| 550 | 26.77 | 20.39 | 25.56 | 20.34 |
| 551 | 29.26 | 26.34 | 27.78 | 26.01 |
| 552 | 27.18 | 22.70 | 26.44 | 22.46 |
| 553 | 32.19 | 25.85 | 31.52 | 25.76 |
| 554 | 29.75 | 24.20 | 28.39 | 23.64 |
| 555 | 31.74 | 26.50 | 31.17 | 26.45 |
| 556 | 30.82 | 27.17 | 29.95 | 26.81 |
| 557 | 23.89 | 18.53 | 23.18 | 18.26 |
| 558 | 25.14 | 18.85 | 24.06 | 18.43 |
| 559 | 25.64 | 22.66 | 24.24 | 22.27 |
| 560 | 32.12 | 28.68 | 31.14 | 28.52 |
| 561 | 29.84 | 27.13 | 28.96 | 26.76 |
| 562 | 23.99 | 21.49 | 22.59 | 21.21 |
| 563 | 26.48 | 23.86 | 25.10 | 23.53 |
| 564 | 27.63 | 24.43 | 26.46 | 23.95 |
| 565 | 28.33 | 23.33 | 26.94 | 23.03 |
| 566 | 26.49 | 20.71 | 25.39 | 20.67 |
| 567 | 27.95 | 22.09 | 26.50 | 21.91 |
| 568 | 27.45 | 23.24 | 26.09 | 23.23 |
| 569 | 30.47 | 26.73 | 29.85 | 26.68 |
| 570 | 29.12 | 26.16 | 28.38 | 26.12 |
| 571 | 23.56 | 18.38 | 22.49 | 18.25 |
| 572 | 29.22 | 26.12 | 28.49 | 25.92 |
| 573 | 27.81 | 23.59 | 26.60 | 23.09 |
| 574 | 24.19 | 19.14 | 23.25 | 19.09 |
| 575 | 25.51 | 21.58 | 24.73 | 21.37 |
| 576 | 30.13 | 24.54 | 28.86 | 24.35 |
| 577 | 29.23 | 23.26 | 28.26 | 22.85 |
| 578 | 33.41 | 28.19 | 32.30 | 27.97 |
| 579 | 29.49 | 24.86 | 28.38 | 24.58 |
| 580 | 24.79 | 20.17 | 24.24 | 20.10 |
| 581 | 30.99 | 25.63 | 30.23 | 25.38 |
| 582 | 24.87 | 19.66 | 23.76 | 19.62 |
| 583 | 31.81 | 28.70 | 31.04 | 28.56 |
| 584 | 29.58 | 26.58 | 28.63 | 26.43 |
| 585 | 31.54 | 27.63 | 30.47 | 27.39 |
| 586 | 28.87 | 23.73 | 27.65 | 23.19 |
| 587 | 26.90 | 21.54 | 26.28 | 21.43 |
| 588 | 24.45 | 21.12 | 23.91 | 20.99 |
